# Effect of Clockwise Reciprocation Motion of Optimum Torque Reverse Kinematic on the Cyclic Fatigue Resistance of Nickel–Titanium Rotary Instruments with Different Metallurgical Properties

**DOI:** 10.3390/ma19020387

**Published:** 2026-01-18

**Authors:** Jorge N. R. Martins, Emmanuel J. N. L. Silva, Duarte Marques, João Caramês, Francisco M. Braz Fernandes, Marco A. Versiani

**Affiliations:** 1 Faculdade de Medicina Dentária, Universidade de Lisboa, 1600-277 Lisboa, Portugal; 2 LIBPhys-FCT UID/FIS/04559/2020, Universidade de Lisboa, 1600-277 Lisboa, Portugal; 3 Grupo de Investigação em Bioquimica e Biologia Oral (GIBBO), Unidade de Investigação em Ciências Orais e Biomédicas (UICOB), 1600-277 Lisboa, Portugal; 4 Centro de Estudos de Medicina Dentária Baseada na Evidência (CEMDBE), 1600-277 Lisboa, Portugal; 5 Department of Endodontics, School of Dentistry, Grande Rio University (UNIGRANRIO), Rio de Janeiro 21210-623, Brazil; 6 Department of Endodontics, Fluminense Federal University, Rio de Janeiro 24220-900, Brazil; 7 Department of Endodontics, Rio de Janeiro University (UERJ), Rio de Janeiro 20550-013, Brazil; 8 CENIMAT/I3N, Department of Materials Science, NOVA School of Science and Technology, Universidade NOVA de Lisboa, 2829-516 Caparica, Portugal; 9 Dental Specialty Center, Brazilian Military Police, Belo Horizonte 30350-190, Brazil

**Keywords:** cyclic fatigue, differential scanning calorimetry, endodontics, kinematics, nickel-titanium instruments, reciprocation, root canal therapy

## Abstract

This study evaluated the effect of clockwise reciprocation motion used in the original Optimum Torque Reverse kinematics, compared with clockwise continuous rotation, on the cyclic fatigue strength of nickel–titanium rotary instruments (NiTi) with different metallurgical characteristics. A total of 120 instruments, ProFile and EndoSequence in sizes 25/.04, 30/.04, and 35/.04, were tested under continuous rotation or reciprocation motions (n = 10 per subgroup). Instruments were examined by optical and scanning electron microscopy to exclude manufacturing defects. Phase transformation temperatures were determined by differential scanning calorimetry, and cyclic fatigue testing was conducted using a custom device simulating a curved canal with a 6 mm radius and an 86° curvature. The time to fracture was recorded, and the number of cycles to fracture was calculated. Statistical comparisons were performed using the Mann–Whitney U test with a significance level of *p* < 0.05. Differential scanning calorimetry showed that ProFile instruments were fully austenitic at the test temperature, while EndoSequence instruments exhibited a mixed R-phase and austenitic structure. Clockwise reciprocation motion significantly increased cyclic fatigue resistance in all groups compared with clockwise continuous rotation. Time to fracture increased by 241.3% to 337.5%, and EndoSequence instruments consistently demonstrated higher fatigue resistance. The greatest relative improvement was observed in ProFile 35/.04, with a 422.4% increase in the number of cycles to fracture. Overall, the reciprocation motion markedly enhanced cyclic fatigue strength irrespective of metallurgical phase composition, indicating a practical mechanical benefit that may reduce the risk of instrument separation during endodontic procedures.

## 1. Introduction

Nickel–titanium (NiTi) rotary instruments have changed endodontic practice by improving shaping the efficiency, flexibility, and preservation of canal anatomy [1]. Their superelasticity and shape memory allow for the safer and more consistent preparation of complex root canal systems than stainless-steel instruments [1]. However, unexpected instrument fracture remains a clinically relevant complication that can compromise treatment outcomes [2]. Instrument separation primarily occurs through cyclic fatigue and torsional failure [2,3]. Cyclic fatigue results from repeated tensile and compressive stresses in curved canals, leading to crack initiation and propagation without visible warning [3,4]. Torsional failure occurs when the instrument tip binds within the canal while the shank continues to rotate, generating stresses that exceed the elastic limit of the alloy [3]. To reduce these risks, advances in heat treatment, instrument design, and kinematic strategies have been introduced.

Reciprocating motion represents a major kinematic development, particularly for improving resistance to cyclic fatigue [5]. Alternating clockwise and counterclockwise movements reduce continuous rotational stress and disrupt crack propagation, thereby extending fatigue life. More recently, Optimum Torque Reverse (OTR) kinematics were introduced for instruments originally designed for continuous rotation [6]. This system combines real-time torque monitoring with adaptive motion. Under low load, the instrument rotates continuously, and when torque exceeds a preset threshold, it switches to reciprocation, reducing the stress concentration while maintaining cutting efficiency.

Metallurgical properties also critically influence instrument performance. Depending on heat treatment, NiTi instruments may present different phase distributions at clinical temperatures, ranging from fully austenitic structures to mixtures containing R-phase or martensite, which directly affect the flexibility and mechanical behavior [7,8]. Although OTR motion, with its clockwise reciprocation phase, has shown promising results in improving the performance of rotary instruments [9,10], it remains unclear whether its clockwise reciprocation kinematics provide similar benefits for instruments of identical size and taper but with different crystallographic arrangements, as determined by phase transformation analysis. Understanding the interaction between kinematics and phase transformation behavior may guide more rational instrument selection, particularly in complex anatomies.

Therefore, the aim of this study was to evaluate the effect of clockwise reciprocating kinematics, compared with clockwise continuous rotation, on the cyclic fatigue strength of NiTi rotary instruments with an identical size and taper but different metallurgical characteristics. The null hypothesis was that there would be no difference in cyclic fatigue strength between clockwise continuous rotation and clockwise reciprocating motion within any instrument group.

## 2. Materials and Methods

### 2.1. Sample Selection

**Figure 1:**
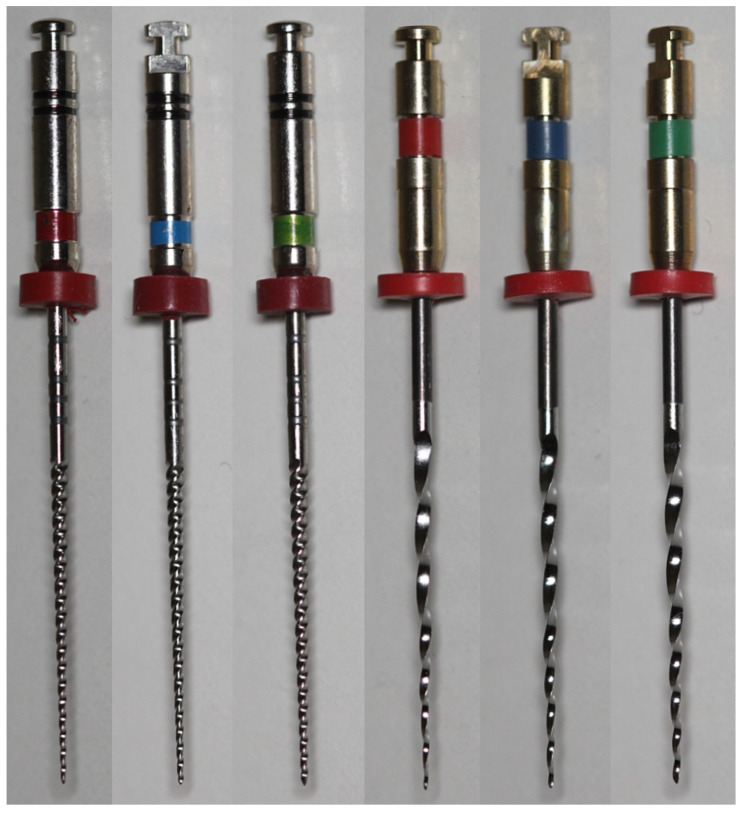
Representative images of the tested instruments. From left to right: ProFile 25/.04, 30/.04, and 35/.04 and EndoSequence 25/.04, 30/.04, and 35/.04.

One hundred and twenty new 25 mm ProFile 25/.04, 30/.04, and 35/.04 (Dentsply Maillefer, Ballaigues, Switzerland) and EndoSequence 25/.04, 30/.04, and 35/.04 (Brasseler USA, Savannah, GA, USA) rotary instruments were used in this study (Figure 1) (n = 20 per group). Instruments were randomly selected and examined under a dental operating microscope at 13.6× magnification (Opmi Pico, Carl Zeiss Surgical, Jena, Germany) to identify major structural irregularities. The instruments (n = 3 per group) were then mounted in a holder and evaluated by scanning electron microscopy (S-2400, Hitachi, Tokyo, Japan) to characterize machining-related surface features and detect minor manufacturing defects. No instruments were excluded based on these evaluations.

### 2.2. Differential Scanning Calorimetry Analysis

Differential scanning calorimetry was performed using a DSC 204 F1 Phoenix system (Netzsch-Gerätebau GmbH, Selb, Germany) to determine the phase transformation temperatures of ProFile 30/.04 and EndoSequence 30/.04 instruments, which served as representatives of their respective systems. The procedure followed ASTM F2004-17 guidelines [11]. For each instrument, a 4 to 5 mm segment weighing 5 to 10 mg was cut from the active blade and immersed for 2 min in an etching solution containing 45% nitric acid, 25% hydrofluoric acid, and 30% distilled water. The specimens were then rinsed with distilled water and placed in aluminum pans for analysis, with an empty pan used as the baseline. The thermal program lasted 1 h and 40 min and was conducted under a nitrogen atmosphere. Temperature was cycled from −150 °C to 150 °C at a constant rate of 10 °C per minute. Data acquisition and processing, including generation of transformation temperature curves, were performed using Netzsch Proteus Thermal Analysis software (v.7.1; Netzsch-Gerätebau GmbH, Selb, Germany).

### 2.3. Mechanical Strength

Sample size calculation was determined through a priori power analysis based on preliminary comparisons (6 initial tests) between continuous rotation and reciprocating motion (using the OTR mode). Assuming a significance level of 0.05 and a statistical power of 80%, and using the following mean values with their respective standard deviations as estimated effect sizes, 66.0 ± 36.4 (ProFile 25/.04), 51.8 ± 26.7 (ProFile 30/.04), 57.8 ± 29.3 (ProFile 35/.04), 103.4 ± 53.3 (EndoSequence 25/.04), 92.8 ± 46.8 (EndoSequence 30/.04), and 82.6 ± 43.3 (EndoSequence 35/.04), the minimum required sample size was six instruments per group. To improve reliability and maintain adequate statistical power, ten instruments per group were included.

Cyclic fatigue testing was performed using a custom-built device that allowed free rotation of the instruments inside a simulated curved canal until fracture. The artificial canal consisted of a non-tapered stainless-steel tube, 19 mm in length, divided into three segments: a 7 mm straight coronal portion, a 9 mm curved middle portion with a 6 mm radius and an 86° curvature, where maximum stress occurred at the midpoint, and a 3 mm straight apical portion, as used in previous studies [3,9]. The tube had an internal diameter of 1.4 mm. The canal block was mounted on a main frame with a mobile handpiece holder to ensure accurate and reproducible instrument positioning at a constant working length. Within each group, instruments were randomly assigned to two subgroups (n = 10): continuous rotation and reciprocating motion. All instruments were operated using the E-connect S endodontic motor (Eighteeth, Changzhou, China) according to the manufacturers’ recommendations.

In the continuous rotation subgroup, ProFile instruments were operated at 250 rpm and 3.0 N·cm and EndoSequence instruments at 500 rpm and 2.5 N·cm, with auto-stop and auto-reverse functions disabled. In the reciprocating motion subgroup, instruments were operated in reciprocating mode at 300 rpm with 150° clockwise and 30° counterclockwise movements, also with auto-stop and auto-reverse disabled (keeping only the fixed-angle clockwise reciprocating motion). All tests were performed at room temperature (20 °C) with lubricant (glycerin). Each instrument was run until fracture occurred, detected visually or audibly. Time to fracture was recorded in seconds with a digital stopwatch, and the number of cycles to fracture was calculated by multiplying the time to fracture by the operating speed (250, 300 or 500 rpm) and dividing by 60 s.

### 2.4. Statistical Analysis

Time to fracture and number of cycles to fracture were not normally distributed, as confirmed by the Shapiro–Wilk test (*p* < 0.05). Accordingly, group comparisons were performed using the nonparametric Mann–Whitney U test, and results are presented as medians with interquartile ranges. Statistical significance was set at *p* < 0.05 (SPSS v.22; IBM SPSS Statistics, Chicago, IL, USA).

## 3. Results

**Figure 2:**
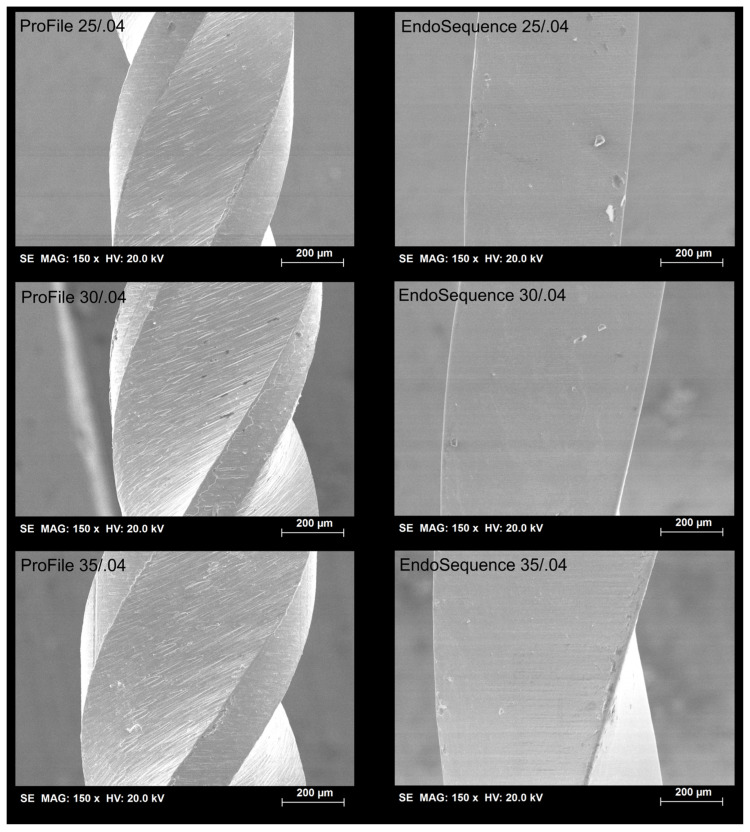
Representative scanning electron microscopy images show the surface morphology of the evaluated instruments’ middle sections.

Microscopic inspection showed no major structural defects or deformations in any instrument before cyclic fatigue testing. Scanning electron microscopy revealed that ProFile instruments had surface finishes with parallel machining marks and occasional minor metal rollovers, consistent with their manufacturing process. In contrast, EndoSequence instruments exhibited a smoother surface topography with fewer irregularities (Figure 2).

**Figure 3:**
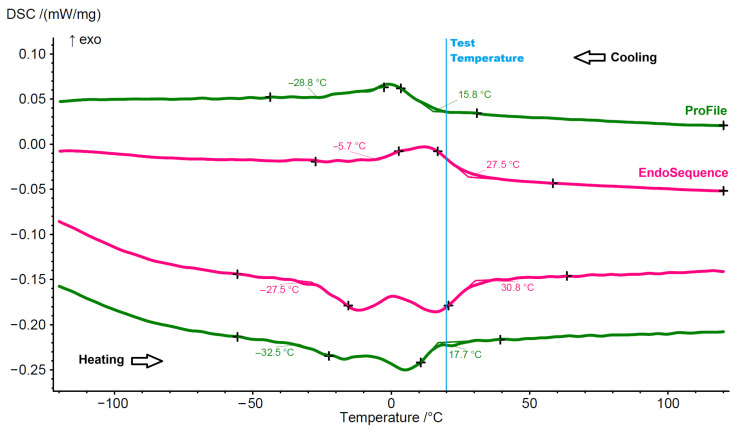
Differential scanning calorimetry diagrams showing the phase transformation temperatures of both instruments. The upper curves represent the cooling cycle, read from right to left, and the lower curves represent the heating cycle, read from left to right.

Differential scanning calorimetry demonstrated distinct phase transformation behavior between the systems. ProFile instruments showed an R-phase start temperature of 15.8 °C and an R-phase finish temperature of −28.8 °C. EndoSequence instruments showed an R phase start temperature of 27.5 °C and an R-phase finish temperature of −5.7 °C. At the test temperature, ProFile instruments were predominantly in the austenitic phase, while EndoSequence instruments exhibited a mixed R-phase and austenitic structure (Figure 3). The austenitic start (As) and finish (Af) for ProFile instruments were −27.5 °C and 30.8 °C, respectively, while for EndoSquence files, they were −32.5 °C and 17.7 °C, respectively (Figure 3).

Cyclic fatigue testing showed a significant increase in fatigue resistance under reciprocation motion compared with continuous rotation (*p* < 0.001 for all comparisons). ProFile 25/.04 instruments fractured after a median of 21.5 s under continuous rotation, increasing to 86.5 s under reciprocation, corresponding to a 302.3% increase. EndoSequence 25/.04 instruments increased from 31 to 128 s, a 312.9% increase. Similar trends were observed in all groups, with improvements ranging from 241.3% to 337.5%. When instruments with identical apical size and taper were compared, EndoSequence instruments consistently showed higher cyclic fatigue resistance than the corresponding ProFile instruments under both kinematics (Table 1).

## 4. Discussion

NiTi mechanical instruments have improved endodontic practice by enabling more predictable shaping of complex root canal systems, while preserving original anatomy with higher efficiency [1,12]. Despite these advantages, unexpected instrument separation remains a relevant clinical problem. When a fracture occurs inside the canal before complete debridement, it can prevent proper cleaning and disinfection procedures. This situation can compromise treatment outcomes and often requires additional procedures such as bypassing or surgical retrieval [13,14]. Versiani et al. [13] investigated this complication and showed that retrieval procedures, although frequently successful, produce biological and structural damage. Their micro-CT analyses demonstrated that even conservative retrieval methods caused significant dentine removal and thinning of the canal walls, especially in narrow and curved areas such as mesial roots of mandibular molars. Ultrasonic techniques removed fragments faster but generated more dentinal thinning than the lasso-assisted approach. These findings indicate that retrieval carries predictable tissue costs, particularly in regions with limited initial dentin thickness. This evidence supports a preventive strategy focused on reducing the risk of instrument fracture rather than relying on post-fracture management.

Cyclic fatigue represents the mechanism of instrument separation resulting from repeated tension and compression stresses as the instrument rotates inside curved canals [3,15,16,17]. For this reason, researchers have focused on strategies that reduce cyclic fatigue. One strategy involves the OTR motion proposal [6]. Unlike continuous rotation, OTR uses a hybrid kinematic pattern. The instrument rotates continuously under low resistance and shifts to reciprocating motion when the torque exceeds a preset threshold. This adaptive behavior reduces sustained mechanical stress and interrupts crack growth, which increases instrument longevity. Based on this principle, the present study evaluated whether clockwise reciprocation motion used in OTR kinematics increased the cyclic fatigue strength of two NiTi rotary systems, ProFile and EndoSequence, that share a similar size and taper but differ in alloy characteristics. The results rejected the null hypothesis, because reciprocating motion significantly increased the fatigue strength in all tested groups.

Both ProFile and EndoSequence instruments showed significant improvements in cyclic fatigue strength when used under reciprocation motion compared with continuous rotation. This effect occurred across all tested sizes, including 25/.04, 30/.04, and 35/.04. The percentage increase in time to fracture ranged from 241.3% to 337.5%, while the increase in the number of cycles to fracture ranged from 104.8% to 422.4% (Table 1). These results agree with the findings of Pedullà et al. [6], who reported that the OTR motion increased cyclic fatigue strength in multiple file systems, including ProTaper Next, Mtwo, and Twisted Files. They attributed this effect to torque controlled switching, which reduces sustained stress and introduces reciprocating phases that interrupt continuous fatigue loading. This mechanism explains the fatigue resistance improvements observed in the present study and supports previous reports [9,10].

The present outcomes add new information by comparing two systems with similar size and taper but different crystallographic structures. ProFile instruments consist of conventional austenitic NiTi. EndoSequence instruments use a proprietary heat treatment and show a mixture of R-phase and austenite at room temperature, which increases flexibility. Despite these metallurgical differences, reciprocating motion increased cyclic fatigue strength in both systems. This finding indicates that the protective effect of OTR applies across different phase compositions and crystallographic arrangements. These findings agree with Martins et al. [10], who evaluated the effect of OTR on ProTaper Next instruments. They reported a significant increase in the time to fracture compared with continuous rotation. Fractographic analysis showed a wider distribution of microcracks under OTR. This pattern supports that the OTR mode distributes mechanical stress more evenly along the instrument, limits local stress concentration, and slows the microcrack growth that usually precedes catastrophic fracture.

The present study also found that EndoSequence instruments showed higher baseline resistance to cyclic fatigue under continuous rotation. However, the relative improvement produced by clockwise reciprocating kinematics was greater in ProFile instruments. For example, ProFile 35/.04 showed a 337.5% increase in the time to fracture and a 422.4% increase in the number of cycles to fracture. In contrast, EndoSequence 35/.04 showed increases of 241.3% and 104.8%, respectively. These results indicate that metallurgy defines baseline mechanical performance, while clockwise reciprocation tends to provide greater benefit to systems with lower inherent cyclic fatigue resistance. This effect may improve their clinical reliability. Taken together, the results show that clockwise reciprocating kinematics improve fatigue performance in NiTi systems, even in instruments not designed for reciprocating use. The improvement occurs regardless of the heat treatment or phase composition.

Although the instruments shared identical apical size and taper, differences in cross-sectional design and manufacturer-recommended motor parameters between systems may influence fatigue behavior; however, the present study did not aim to compare the systems directly but rather to evaluate how clockwise continuous rotation and clockwise reciprocation perform within two distinct crystallographic arrangements under relevant conditions. Nevertheless, the influence of crystallographic arrangement, instrument geometry, and kinematic mode could not be strictly isolated in the present experimental design; therefore, the observed differences should be interpreted as comparative findings within the two tested systems rather than as universal conclusions applicable to all heat-treated NiTi instruments.

Some limitations must be considered before applying these findings in clinical practice. Cyclic fatigue tests used standardized artificial canals with a fixed curvature, fixed radius, and a single controlled temperature. This model improves repeatability but does not reproduce the complexity of natural root canals, where anatomical variability, curvature irregularity, and temperature changes affect instrument behavior. In addition, this study focused only on cyclic fatigue and did not evaluate other mechanical properties or clinical outcomes such as shaping ability, canal transportation, or debris extrusion, which kinematics may also influence. Another practical limitation is that not all endodontic motors currently support the original OTR function. The strengths of this study include the large sample size of 120 instruments, metallurgical evaluation by differential scanning calorimetry, and the testing of three sizes across two systems, which clarifies the interaction between metallurgy and kinematics in fatigue behavior. The use of a lubricant was intended to standardize testing conditions and minimize frictional interference and heat generation during cyclic loading. Nevertheless, the testing environment is a relevant variable in fatigue research, and lubricants may indirectly influence fatigue behavior by modifying frictional conditions and thermal/mechanical stress distribution. Although lubricant was applied uniformly across all groups, and therefore the comparative validity of the results is preserved, its potential contribution to absolute fatigue values cannot be fully excluded.

Future studies should include micro-CT analysis to assess the effect of clockwise reciprocating kinematics on shaping ability and canal preservation. Evaluation of torsional resistance under clockwise reciprocating motion and testing cyclic fatigue across a wider range of clinically relevant temperatures and chemical environments may further validate these findings.

## 5. Conclusions

Clockwise reciprocation motion increased the cyclic fatigue strength of NiTi rotary instruments across their metallurgical characteristics. Both ProFile and EndoSequence systems showed significant increases in time to fracture (which increased by 241.3% to 337.5%) and the number of cycles to fracture (which increased by 104.8% to 422.4%) when operated under reciprocation compared with continuous rotation. These results indicate that this kinematic mode provides a consistent mechanical advantage by reducing stress accumulation and delaying crack initiation and propagation. As a result, clockwise reciprocation has the potential to lower the risk of instrument separation and to improve the mechanical safety of root canal instrumentation in clinical practice.

## Figures and Tables

**Table 1:** Time and number of cycles to fracture (median [interquartile ranges]) and their percentage increases when moving from continuous rotation to reciprocating kinematic.

Instrument	Clockwise Rotation Group	Clockwise Reciprocation Group	Increase	*p*-Value
Time to fracture (seconds)				
ProFile 25/.04	21.5 [19.8–27.3]	86.5 [77.5–97.0]	302.3%	<0.001
ProFile 30/.04	21.5 [17.8–25.0]	75.0 [62.3–82.3]	248.8%	<0.001
ProFile 35/.04	16.0 [13.8–21.0]	70.0 [62.8–76.0]	337.5%	<0.001
EndoSequence 25/.04	31.0 [28.0–38.3]	128.0 [102.3–139.8]	312.9%	<0.001
EndoSequence 30/.04	29.0 [24.5–39.3]	125.5 [116.5–130.5]	332.8%	<0.001
EndoSequence 35/.04	31.5 [22.8–41.8]	107.5 [99.5–123.8]	241.3%	<0.001
Cycles to fracture (number of cycles)				
ProFile 25/.04	90.0 [82.0–113.3]	432.5 [387.5–485.0]	380.6%	<0.001
ProFile 30/.04	90.0 [74.0–104.0]	375.0 [311.3–411.3]	316.7%	<0.001
ProFile 35/.04	67.0 [57.0–88.0]	350.0 [313.8–380.0]	422.4%	<0.001
EndoSequence 25/.04	258.5 [233.0–318.5]	640.0 [511.3–698.8]	147.6%	<0.001
EndoSequence 30/.04	241.5 [204.0–327.3]	627.5 [582.5–652.5]	159.8%	<0.001
EndoSequence 35/.04	262.5 [189.8–348.3]	537.5 [497.5–618.8]	104.8%	<0.001

## Data Availability

The original contributions presented in the study are included in the article. Further inquiries can be directed to the corresponding author.
